# Urban Health Research in Africa: Themes and Priority Research Questions

**DOI:** 10.1007/s11524-016-0050-0

**Published:** 2016-05-16

**Authors:** Tolu Oni, Warren Smit, Richard Matzopoulos, Jo Hunter Adams, Michelle Pentecost, Hanna-Andrea Rother, Zulfah Albertyn, Farzaneh Behroozi, Olufunke Alaba, Mamadou Kaba, Claire van der Westhuizen, Maylene Shung King, Naomi S. Levitt, Susan Parnell, Estelle V. Lambert, RICHE members

**Affiliations:** 1 Division of Public Health Medicine, School of Public Health and Family Medicine, University of Cape Town, Cape Town, South Africa; 2 African Centre for Cities, University of Cape Town, Cape Town, South Africa; 3 Burden of Disease Research Unit, South African Medical Research Council, Cape Town, South Africa; 4 Health Economics unit, School of Public Health and Family Medicine, University of Cape Town, Cape Town, South Africa; 5 Department of Anthropology, University of Cape Town, Cape Town, South Africa; 6 Institute of Social and Cultural Anthropology, University of Oxford, Oxford, UK; 7 Division of Environmental Health and Centre for Environmental and Occupational Health Research, School of Public Health and Family Medicine, University of Cape Town, Cape Town, South Africa; 8 Children’s Institute, Department of Paediatrics, University of Cape Town, Cape Town, South Africa; 9 Primary Health Care Directorate, University of Cape Town, Cape Town, South Africa; 10 Division of Health Economics, School of Public Health and Family Medicine, University of Cape Town, Cape Town, South Africa; 11 Division of Medical Microbiology, Department of Clinical Laboratory Sciences, University of Cape Town, Cape Town, South Africa; 12 Alan J Flisher Centre for Public Mental Health, Department of Psychiatry and Mental Health, University of Cape Town, Cape Town, South Africa; 13 Division of Health Policy and Systems, School of Public Health and Family Medicine, University of Cape Town, Cape Town, South Africa; 14 Chronic Disease Initiative for Africa and Division of Diabetic Medicine and Endocrinology, Department of Medicine, University of Cape Town, Cape Town, South Africa; 15 Division of Exercise Science and Sports Medicine, Department of Human Biology, University of Cape Town, Cape Town, South Africa

## Introduction

In Africa, urbanization and urban growth are dramatically restructuring the nature of cities. The growing majority of urban dwellers now live in informal conditions that, without access to basic services or public amenities, expose residents to greater health risk, and health-care systems are unable to provide affordable or comprehensive cover. The differential exposure to these urban conditions is compounded by social and economic vulnerability, resulting in health inequities. Yet despite pressing needs driven by Africa’s considerable and complex burden of disease and high levels of health inequity, urban health and urban health equity have not yet emerged as major research and policy priorities in Africa, and as such South Africa, like many other African countries, lags behind in addressing these issues.

**FIG. 1:**
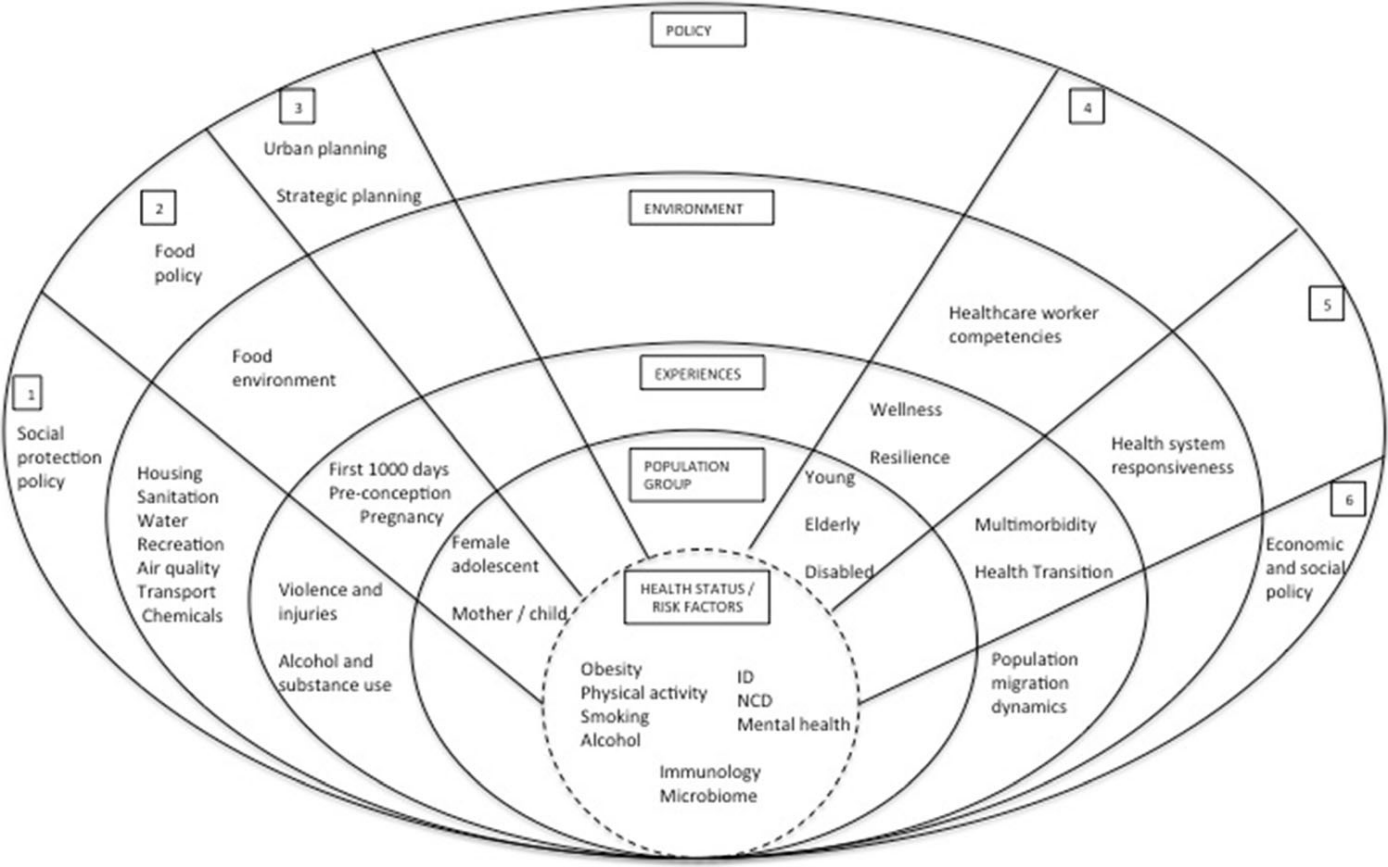
Conceptual framework of public health approach to urban health research themes in Africa.

This commentary presents a conceptual framework, using a public health approach, for interdisciplinary research aimed at contributing to the understanding and mitigation of urban health issues and challenges in Africa (Fig. 1). This approach identifies downstream and upstream factors, based on published literature, associated with key determinants in each theme. In other words, in addition to the individual level risk factors, the figure summarizes factors associated with each theme at the community, experiential, environmental, and structural policy levels. It represents a collective effort by interdisciplinary academics from public health; anthropology; civil engineering; architecture, planning and geomatics; human biology; psychiatry and mental health; medicine; pathology; and paediatrics, from the Research Initiative for Cities and Health (*RICHE*), University of Cape Town (UCT), to generate African perspectives on urban health and urban health equity. A workshop to tackle the urban health research agenda in August 2015 was attended by 40 *RICHE* members with extensive global and local urban health experience. In line with the co-production philosophy advocated for African urban contexts 1, additional contributions came from representatives from the Western Cape Department of Health. The process of identifying themes and gaps was as follows: First existing urban health research in Africa was presented. The workshop participants, who have a wide range of experience in urban health in Africa presented what work was ongoing or planned. Through this iterative process, the key components of research questions identified were then thematically classified into six distinct focus areas. Using a public health, socio-ecological model, these themes were then classified into different levels from the individual to policy levels. Gaps in research were then identified qualitatively by the same iterative process from which unanswered priority research questions were identified. Three cross-cutting principles for African urban health research were also identified in the process, based on methodological and technical requirements for the successful conduct of the proposed research themes (Table 1).

**TABLE 1:** Urban health research focus areas and priority research questions

Focus areas	Urban health priority research questions
1. Obesity and food insecurity nexus	What are the changing patterns of the informal and formal retail food environment?
	How do the food environment and food policies influence population diet, obesity, food insecurity, and health?
	Using a life-course approach, what are the most effective meso- and macro-level interventions to reduce obesity and improve food security (focus on women-headed households, the first 1000 days and female adolescents)?
2. Urban context as a tool for health promotion	Can the housing environment be a tool for health promotion and disease prevention?
	What is the impact of urban regeneration and upgrades to the transport and physical activity/recreational environments on experienced violence/injuries, alcohol or drug use, mental health, and chronic diseases?
	To what extent does community engagement and social mobilization influence urban planning and prioritization with respect to the urban built environment and broader health nexus?
	What role can schools play as potential community hubs of wellness?
	What are the most (cost) effective and contextually relevant environmental interventions to improve health through the control and prevention of infectious and zoonotic disease transmission in informal urban settings?
	How can the risks of climate change be mitigated and communicated to a marginalized population?
3. Urban health governance and policy	What is the influence of urban population dynamics in the implementation of innovative urban health and economic policy interventions and programs within the context of equity?
	What is the impact of incorporating health objectives in strategic spatial planning (as part of a “Health in All Policies” approach) on health and well-being?
	What are the barriers and facilitators to policy reform towards health in all policies?
4. Community strengthening for healthy inclusive cities	How do we best measure/quantify community wellness and resilience, using participatory methods and routine administrative data to develop indicators/indices (for general and vulnerable urban populations: youth, disabled, elderly)?
	How can communities work together with the natural resource of an increasingly aging population to address social isolation, access and vulnerability?
	What core competencies are needed for health workers to assist with negotiating and advocating for disability-inclusive and environmentally just community development?
	Can community strategies that focus on increasing engagement among youth with respect to health and environmental literacy/risk communication and a community health needs assessment, influence risk- and health-seeking behavior in the youth?
5. Health systems in an urbanizing context	How can health systems better respond to changing patterns of disease in urban settings to address children and adults with chronic infectious, mental health, and NCD multi-morbidity?
	How can health systems and policies incorporate environmental health concerns into mainstream health policies (e.g., climate change, air pollution, chemical poisonings)?
6. Migration, urbanization and health	What are the patterns of urban population dynamics (intra-urban and rural-urban population circulation) and can exposure to key aspects of urban environment (including pathogens, air, food, vectors, chemicals) be quantified and measured spatio-temporally?
	Given the dynamic nature of urban exposure, how does exposure to the urban environment influence the inflammatory status, microbiome, and ultimately biopsychosocial health (physical activity, obesity, alcohol or drug use, diabetes, hypertension/cardiovascular disease, HIV, tuberculosis, mental disorders, depression, social cohesion, resilience) of urban residents?

## Six Focus Area Themes

### Urban Context as a Tool for Health Promotion and Disease Prevention

The health and well-being of urban residents is intricately linked to the natural, built, and institutional elements of the urban context. As such, all dimensions of urbanization and urban living, including socio-environmental determinants of health need to be considered 2. The Commission on the Social Determinants of Health 3 and the *Hidden Cities*^4^ reports identify addressing urban health as a key priority in the global South. Thus, investigation of the interaction between health and the urban determinants of health (social and policy environments, health services, the built, physical housing and recreational environments, food systems, crime/security, access to potable water, air quality, transport, vector and pest management, community resilience, psycho-social support structures) is essential 5. Understanding these interactions could facilitate developing, implementing, and evaluating interventions that aim to harness these aspects of the urban context as tools for health promotion and prevention of infectious and vector-borne diseases, non-communicable diseases, and injuries, which in South African cities account for 20 % more mortality than in rural areas 6. This understanding could be gained via research paired with “natural experiments” and randomized evaluations, such as urban upgrades, housing developments, and integrated transport system routes, to assess the impact on health behaviors and health outcomes.

### Obesity and Food Insecurity Nexus

As in South Africa, the most urbanized African country, many low- and middle-income countries, urban populations on average have better access to health services and are associated with better health indicators overall 7,8. However, urban areas are characterized by significant spatial and environmental deprivation and discrimination, the co-existence of chronic communicable and non-communicable diseases along with a high prevalence of obesity in predominantly food insecure communities 9,10. As urbanization has a direct effect on food systems and therefore nutrition, knowledge of local urban food systems and food ways, including the informal and formal sectors, is needed to address the dual burden of obesity and food security. In particular, given the higher prevalence of obesity in women in many African countries, the importance of pre-conception and peri-partum nutrition on fetal health and the subsequent health of the child into adulthood, research in this area should utilize a life-course approach to more effectively target nutritional interventions for health promotion and disease prevention.

### Urban Health Governance and Policy

There is a growing recognition of the importance of social determinants of health and the need for a multisectoral effort to effectively address these determinants to improve health equity. Yet a coherent strategy has not been implemented in many African countries, resulting in a lack of understanding of the processes in place in different sectors, an absence of a shared learning space, and a paucity of data on the health impact of intersectoral policies and programs. While a number of government departments in South Africa have begun to include health objectives in their strategic and developmental planning 11, the process and impact of this change has not been evaluated. This evaluation and an investigation into the barriers and facilitators to such policy reform are urgently needed. One approach that may guide research in this area is the Health in All Policies (HiAP) approach. This approach recognizes the important roles that structural, environmental, and socio-economic factors play in population health and highlights the need for intersectoral approaches to foster healthy communities and environments. Incorporating the context of informal housing into urban health governance research is required as housing policies of some African countries focus on upgrading, not eliminating, informal settlements. Key elements of this approach 12 include stakeholder (community, private sector, funders, policy makers) engagement, creation of co-benefits for multiple partners and efficiencies across agencies and sectors, and the creation of structural and procedural change to institutionalize HiAP within government processes and initiatives. These elements will support deep and sustainable intersectoral collaboration and promote health and equity in all government policies and programs.

### Community Strengthening for Healthy Inclusive Cities

A healthy city has been defined as one that is “continually creating and improving those physical and social environments and strengthening those community resources which enable people to mutually support each other in performing all the functions of life and achieving their maximum potential” 13. In a major step forward, the new global sustainable development goals recognize the need to make cities inclusive, safe, resilient, and sustainable 14. This goal is especially pertinent for vulnerable population groups (impoverished, low educational attainment, elderly, disabled, young) who are least equipped to compensate for the conditions that epidemiological, nutritional, climate, and urban transitions generate 15,16. This “inverse care law” 17 results in social patterning, not only for access to health care but also “deprivation amplification” with a differential uptake and appropriation of messages promoting health. There is, therefore, a need to understand community well-being and how it affects individual health and to explore resources and positive attributes within communities, as well as health system competencies that can be strengthened and harnessed to improve urban community well-being and inclusivity.

### Health Systems in an Urbanizing Context

Health systems are complex and require consideration of multiple components, factors, actors, and the interrelationships between these in order to adequately address health needs. In the context of urbanization and epidemiological transition in Africa 18, a deeper understanding of how health systems may need to transform in order to muster an appropriate and sustainable response is required. In particular, account must be taken of the co-existence of chronic infectious disease (ID) epidemics such as HIV, emerging chronic non-communicable disease (NCD) epidemics, including mental disorders, and an increasing burden of violence and injuries 19,20. While the South African health system is moving towards a decentralized, integrated approach to primary healthcare, many gaps in implementation exist 21. Furthermore there is a lack of understanding of the needs of dynamic communities and very little community involvement in health-care organization and access planning 22,23. The formal health systems also need to take into consideration the informality that characterizes many African urban settings in order to evaluate how best to reach those most in need. There is therefore a need for context-specific, integrated systems research to enable the health system to be responsive to population health transitions. In addition, it is important to recognise that the health system is part of a broader social system, and the interrelationship between the health and other social systems in mitigating the health needs is of paramount importance.

### Migration, Urbanization, and Health: the Pathways and Mechanisms through Which Exposures to the Urbanizing Environment Influences Health, Health Risk, and Health Outcomes

Relatively high levels of impermanence and mobility characterize the urban population in many African countries 24, particularly in informal areas where circular rural-urban and urban-urban movement is common. However, there is little quantification and appreciation of the nature of these population dynamics over space and time. Consequently, given the often-stark differences in environmental exposures, including pathogens, air, chemicals, and food, between rural and urban communities, a better understanding of urban population dynamics would facilitate investigation of the mechanisms through which these environmental exposures over the life course influence the biopsychosocial health of urban residents. For healthy migration, there is a need to better understand patterns of circular migration and differential health exposures in each stage of migration, as well as the ways in which health, economic, and social policies and systems best respond to mobility.

## Three Cross-Cutting Principles

Cross-cutting principles necessary for these issues to be addressed underpin these themes.

### Integration of Urban Intelligence and Surveillance

Upstream determinants of health (including social protection, employment (formal and informal), education, forensic pathology) need to be monitored by integrating routine datasets across sectors and a coordinated analysis of intersectoral data to inform research and policy priorities and interventions. In South Africa, there are three rural but no urban health and demographic surveillance sites. Indeed, there are currently only two urban surveillance sites in Africa (Nairobi, Kenya and Ouagadougou, Burkina Faso). Integration and coordination of urban intelligence through a surveillance system would contribute vital information that supports the conduct of implementation research and the assessment of natural experiments, to address key societal challenges in South Africa and the African continent. The extent and nature of the urban health indicators surveyed should be based on known health equity gaps and in particular, those that are mutable and that will be responsive to policy and program solutions 25.

### Systems Approach to Monitoring, Impact, and Policy Evaluation

The support, tracking, and evaluation of diverse health and health-related policy and system changes should use a variety of analytical frameworks and approaches, including economic analyses. Context is the key, as current conditions are created by politics and systemic inequalities in society.^26^

### Communication, Dissemination, and Meaningful Translation

For research to improve health disparities for the urban poor, there is a need to promote engaged scholarship and an engaged society with key stakeholder (governmental departments and communities) collaboration to identify research priorities and programs. Innovative knowledge creation, dissemination in public media, and meaningful engagement with broader society are also required to promote health and well-being through embracing principles of citizen science and the adoption of an inclusive, all-of-society, approach.

## Priority Research Questions

Guided by these themes, an urban health research gap analysis was conducted, identifying key gaps in research. Based on these research gaps, we proposed urban health research questions that should be prioritized in Africa (Table 1).

## Conclusion

The themes and cross-cutting principles presented overlap and interact with each other. As such, a complex systems approach is required to investigate and improve understanding of health and well-being in a changing urban context with a view to developing sustainable and cost-effective interventions. This approach:

- Acknowledges the different dimensions of determinants that influence health;
- Understands the importance of addressing gaps in data and access to information from across these dimensions;
- Identifies the need to address inequity in health and health determinants;
- Recognizes the need to contextualize the dimensions of the urban environment and engage all relevant stakeholders across sectors to effectively prioritize intersectoral interventions to improve health; and
- Emphasizes that action to address health determinants should not be solely undertaken by the health sector but requires an interdisciplinary approach and the involvement of key sectors including economic and social development, housing, and planning.

*RICHE* researchers are optimistic that the funding environment will support the need for a public health approach to urban health research themes addressing the African context so as to reduce urban health disparities and promote evidence-based policy making.
